# Primary Neuroendocrine Tumor of the Breast: A Rare Case

**DOI:** 10.1155/cris/5595521

**Published:** 2025-04-21

**Authors:** She's Magnolia C. Ycong, Hannah Lois R. Kangleon-Tan, Kristoff Armand E. Tan

**Affiliations:** Department of Surgery, University of Cebu Medical Center, Mandaue City, Cebu, Philippines

**Keywords:** case report, hormonal therapy, neuroendocrine breast cancer (NEBC), tumors

## Abstract

Primary neuroendocrine tumors (NETs) predominantly affect postmenopausal women. This case study focused on a 54-year-old woman who presented with a painless right breast lump. While the lump exhibited estrogen and progesterone receptor (PR) positivity, it lacked human epidermal growth factor receptor 2 expression. Further evaluation revealed positivity for the neuroendocrine markers chromogranin A (CGA) and synaptophysin (SYN). It also revealed a 3% positive Ki-67 proliferation index. Treatment for neuroendocrine breast cancer (NEBC) mirrors that of standard invasive breast cancer: breast conservation or mastectomy combined with sentinel lymph node biopsy or axillary dissection. The patient underwent a right mastectomy with sentinel lymph node biopsy, followed by hormonal therapy based on her tumor's immunohistochemical profile. Due to the low incidence and limited research on primary NETs, their exact origin remains shrouded in mystery. Accurate diagnosis, specific treatment options, and long-term prognosis remain significant challenges in managing this rare form of breast cancer.

## 1. Introduction

Neuroendocrine neoplasms (NENs) of the breast are an uncommon and a poorly defined type of breast cancer [1]. The World Health Organization (WHO), in its 2019 classification of breast cancer, explained that NENs of the breast accounts for a variable incidence ranging from <0.1% to as high as 20% [2]. This case is of a postmenopausal woman who presented with a firm painless lump on the right breast, and upon workup, she was found to have a neuroendocrine tumor (NET) of the breast. This work is reported in line with the SCARE 2020 criteria [3].

## 2. Case Report

A 54-year-old, postmenopausal Filipino woman presented at the clinic with a gradually enlarging painless lump on the right breast, associated with an episode of bloody nipple discharge 3 months prior to consult. She had no family history of breast cancer, no previous breast surgeries, nor had any allergies. Her physical exam revealed a firm nontender mass at the 10 o'clock position on the right breast just above the nipple–areola complex with no nipple discharge at the time of examination. The axillary nodes on the right were not clinically palpable.

**Figure 1:**
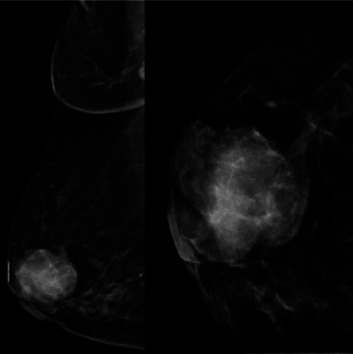
Mammography views: craniocaudal (CC) and mediolateral oblique (MLO) views of the right breast showing a hyperdense lesion with no microcalcification nor architectural distortion at 10 to 12 o'clock positions at the periareolar area.

**Figure 2:**
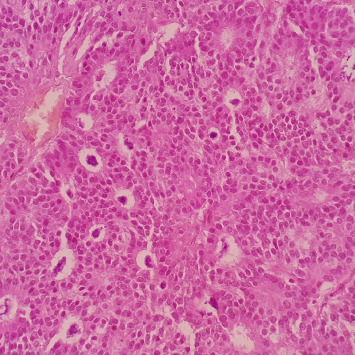
Core needle biopsy showing atypical polygonal to columnar cells arranged in nests, sheets, and rosettes, with attempts at papillary formation and supported by occasional fibrovascular to fibrohyalinized stroma.

**Figure 3:**
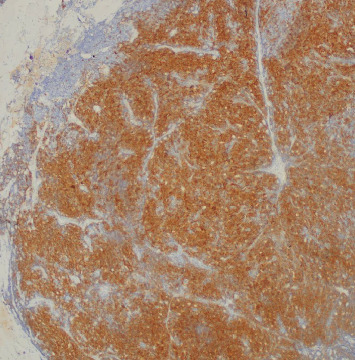
Synaptophysin stain: positive—focal and moderate to strong cytoplasmic granular expression in the neoplastic cells.

**Figure 4:**
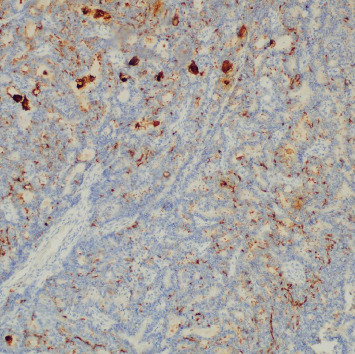
Chromogranin stain: positive—diffuse and strong cytoplasmic expression in neoplastic cells.

The patient's breast workup included a breast ultrasound which showed a 3.6 × 2.8 × 2.7 cm lobulated complex mass, predominantly solid with heterogenous echo pattern at the 10–12 o'clock position at the periareolar area (BI-RADS 4B) and a mammogram with tomosynthesis which demonstrated the hyperdense lesion at the said area with no microcalcification nor architectural distortion (BI-RADS 4B) (Figure 1). The axillary nodes were unremarkable on both imaging studies. A core biopsy of the mass showed atypical polygonal to columnar cells arranged in nests, sheets, and rosettes with attempts at papillary formation and supported by occasional fibrovascular to fibro-hyalinized stroma and no significant mitotic activity (Figure 2) which suggested a NET. Further immunohistochemical staining showed positivity for synaptophysin (SYN) (Figure 3) and chromogranin (Figure 4) which confirms the initial diagnosis. Additionally, it tested positive for hormone receptors, estrogen, and progesterone and negative for human epidermal growth receptor 2. Proliferative index by Ki-67 was low at 3%. Although the patient did not undergo gastroscopy, colonoscopy, and ocreotide scan, further examination yielded no extramammary tumor, particularly in the chest and the gastrointestinal tract by a multislice contrast computed tomography of the chest and abdomen.

**Figure 5:**
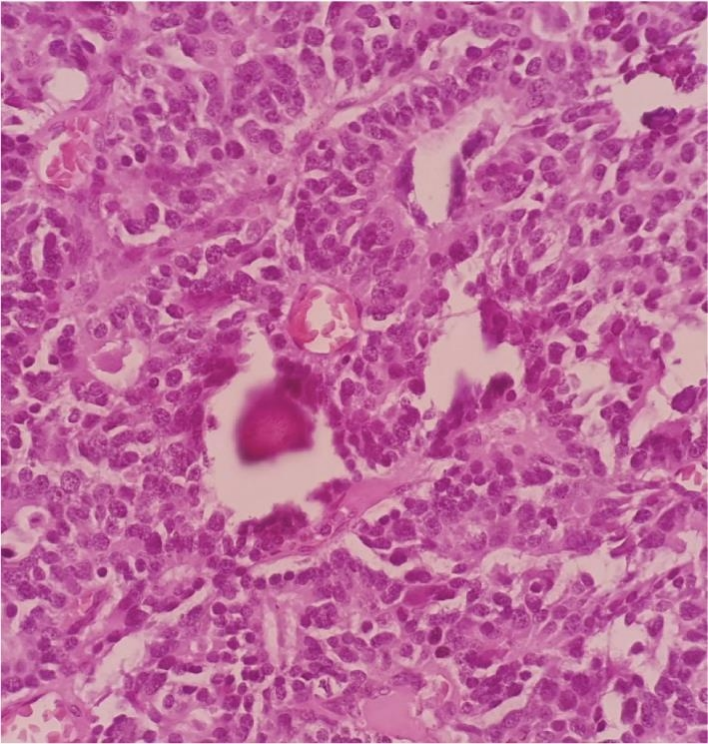
Microscopic findings described as no definitive cell necrosis, significant mitotic activity, lymph vascular and perineural invasion, and involvement of the lactiferous ducts.

**Figure 6:**
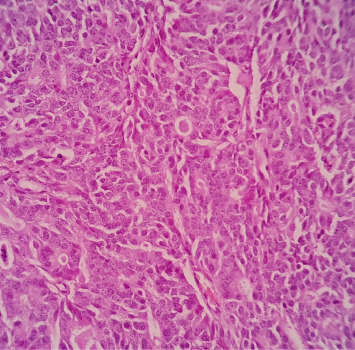
Microscopic finding of the right breast mass, showing nuclei with speckled/salt and pepper chromatin and granular eosinophilic cytoplasm.

The patient was presented with two treatment options: breast conserving surgery with adjuvant radiation or a mastectomy. She elected to undergo the latter approach with sentinel lymph node biopsy. The rest of the hospital course was unremarkable. Final histopathology showed a 3.5 × 2.6 cm well-differentiated NET and negative sentinel nodes. On a microscopic view of the tumor, it showed no definitive cell necrosis and no significant mitotic activity and lymphovascular and perineural invasion, with involvement of the lactiferous ducts (Figure 5). It also showed the nuclei with the characteristic speckled salt and pepper chromatin and granular eosinophilic cytoplasm with rare mitotic activity estimated range from 0 to 1 per 10 high power fields (Figure 6). All margins of resection were clear. She was then maintained on tamoxifen 20 mg once a day based on her breast biomarker results and was on constant follow-up with her oncologist and breast surgeon. Routine mammography on patient follow-up noted no recurrence of the tumor. Patient is in a disease-free state for 5 years.

## 3. Discussion

NEN constitutes all tumor classes with predominant neuroendocrine differentiation, including both well-differentiated and poorly differentiated forms. Mammary-origin NENs comprise less than 1% of NENs and a variable incidence ranging from 0.1% to 20% among breast cancers [1, 2]. Primary neuroendocrine breast tumors is a diagnosis of exclusion and should rule out alternative primary sites. There is no specific clinical or radiological sign to diagnose a neuroendocrine carcinoma (NEC); a histological examination is the only way to confirm the diagnosis of this tumor [4]. These tumors, believed to arise from the endocrine differentiation of breast carcinoma, are classified into three subtypes by the 2012 WHO classification [5]. A 2019 revision aimed for a unified classification, defining NEN as a term encompassing all tumor classes with predominant neuroendocrine differentiation. Furthermore, it subclassified them as well-differentiated, including low- and intermediate-grade tumors (NETs), highly aggressive NECs, and breast carcinoma of no special type (IBCs-NST) with neuroendocrine differentiation. The terminology NENs was introduced, including tumors with prominent neuroendocrine differentiation (presence of histologic neuroendocrine features in more than 90% of the tumor cells). In addition, if neuroendocrine biomarker expression or histological features make up ≤90% of the tumor area, it is defined as an IBC-NST with neuroendocrine features [6].

A study done by Kawasaki et al. noted bloody nipple discharge, although not sufficiently investigated, as an important clinical symptom, especially in breast cancers. They concluded that neuroendocrine ductal carcinoma in situ (NE-DCIS), a distinctive variant of DCIS, is considered to be a preinvasive counterpart of breast NETs. Their study showed that 24 out of 89 patients presented with a bloody nipple discharge and are all histopathologically diagnosed NETs. They speculated the characteristic bloody nipple discharge is attributable to intraductal hemorrhage in association with the vascular structures peculiar to the NE-DCIS components. This component is consistent with the primary nature of the tumor [7]. Bilateral breast carcinoma accounts for ~5% of all patients with breast cancer, while neuroendocrine breast carcinomas comprise less than 5% of invasive breast carcinomas. Therefore, bilateral primary breast NEC is extremely rare [8].

Another study by Sun et al.[9] noted the presence of bloody nipple discharge as one of the main clinical features of neuroendocrine breast cancer (NEBC), which are similar to those of IBS-NST. Compared to invasive ductal cancers of no special type (IDCs-NST), NEBC is more likely to present with distant metastasis at time of diagnosis. In addition to clinical features, most NEBC patients show positive estrogen receptor (ER) and/or progesterone receptor (PR) expression, implying that NECB is part of the luminal-like type. There are certain differences among NEBC, IBC-NST, and IDC-NST in terms of morphological features, and the diagnosis of NEBC is made by histology and IHC staining of neuroendocrine markers. Metastasis from other primary sites to the breast can be excluded by suitable methods, such as chest, abdominal, and pelvic computed tomography scans [9].

Macroscopic examination reveals primary NECs of the breast as round or multilobulated, yellowish colored, with a firm consistency [10]. The gold standard for diagnosis involves immunohistochemical analysis of neuroendocrine biomarkers. With the advent of the IHC techniques, with chromogranin A (CGA) and SYN as the most sensitive and specific markers, it is possible to identify the neuroendocrine phenotypes in this breast cancer subpopulation [11]. In this case, the patient tested positive for both markers, along with estrogen and progesterone hormone receptors [12].

Limited evidence exists for treating NETs, emphasizing the importance of considering prognostic or predictive factors before initiating treatment [13]. Surgical interventions, predominantly mastectomy, axillary dissection, and metastasectomy, constitute the primary treatment approach [14]. Mastectomy is often preferred due to the potentially aggressive nature of breast neuroendocrine neoplasias (Br-NENs) [15].

Chemotherapy and radiotherapy indications align with other breast cancers, considering clinicopathologic factors identified through immunohistochemical staining for ER, PR, HER2, and Ki67 index [16]. Additionally, aromatase inhibitor agents target the mammary component, while anthracycline-based chemotherapy controls the neuroendocrine component [17].

Prognosis data for these heterogeneous tumors are conflicting due to their rarity and changing classification criteria. However, breast primary NETs show a relatively better prognosis than other high-grade NENs, with a 5-year survival exceeding 80% [18]. Key prognostic factors include age, tumor secretion capacity, tumor size, and thepresence or absence of distant metastases [19]. Due to the low documented incidence, there is no specific follow-up guideline. Its follow-up is tailored similar to invasive breast cancer which includes history and physical exam one to four times a year for 5 years then annually thereafter and a screening mammography annually. Postsurgical management includes patient education, referral for incidence of lymphedema, screening for metastasis, and medication adherence [20].

## 4. Conclusion

Breast tumors with neuroendocrine differentiation represent a rare and heterogeneous group, often sharing histological features with invasive breast cancers. Currently, there is lack of specific criteria for its definition and limited evidence to guide specific treatment strategies for breast cancers with neuroendocrine differentiation. Further research is essential to define and categorize this tumor group and establish effective management approaches. This case contributes valuable insights to the limited literature on NENs of the breast, emphasizing the importance of individualized treatment in achieving a favorable outcome.

## Data Availability

Data are available and may be accessed at the University of Cebu Medical Center, Medical Records section, upon reasonable request.
